# New Class of Anti-Inflammatory Therapeutics Based on Gold (III) Complexes in Intestinal Inflammation–Proof of Concept Based on In Vitro and In Vivo Studies

**DOI:** 10.3390/ijms22063121

**Published:** 2021-03-18

**Authors:** Julia B. Krajewska, Jakub Włodarczyk, Damian Jacenik, Radzisław Kordek, Przemysław Taciak, Remigiusz Szczepaniak, Jakub Fichna

**Affiliations:** 1 Department of Biochemistry, Faculty of Medicine, Medical University of Lodz, 92-215 Lodz, Poland; julia.krajewska@stud.umed.lodz.pl (J.B.K.); jakub.wlodarczyk@stud.umed.lodz.pl (J.W.); 2 Department of Cytobiochemistry, University of Lodz, 90-236 Lodz, Poland; damian.jacenik@biol.uni.lodz.pl; 3 Department of Pathology, Faculty of Medicine, 92-213 Lodz, Poland; radzislaw.kordek@umed.lodz.pl; 4 Department of Pharmacodynamics, Faculty of Pharmacy, Medical University of Warsaw, 02-097 Warsaw, Poland; przemyslaw.taciak@wum.edu.pl; 5 Inwex Ltd., 25-323 Kielce, Poland; remigiusz.szczepaniak@inwex.pl

**Keywords:** gold (III) complex, colitis, inflammatory bowel diseases, RAW264.7 macrophages

## Abstract

Inflammatory bowel diseases (IBD) are at the top of the worldwide rankings for gastrointestinal diseases as regards occurrence, yet efficient and side-effect-free treatments are currently unavailable. In the current study, we proposed a new concept for anti-inflammatory treatment based on gold (III) complexes. A new gold (III) complex TGS 121 was designed and screened in the in vitro studies using a mouse macrophage cell line, RAW264.7, and in vivo, in the dextran sulphate sodium (DSS)-induced mouse model of colitis. Physicochemical studies showed that TGS 121 was highly water-soluble; it was stable in water, blood, and lymph, and impervious to sunlight. In lipopolysaccharide (LPS)-stimulated RAW264.7 cells, the complex showed a potent anti-inflammatory profile, as evidenced in neutral red uptake and Griess tests. In the DSS-induced mouse model of colitis, the complex administered in two doses (1.68 μg/kg, intragastrically, and 16.8 μg/kg, intragastrically, once daily) produced a significant (* *p* < 0.05) anti-inflammatory effect, as shown by macroscopic score. The mechanism of action of TGS 121 was related to the enzymatic and non-enzymatic antioxidant system; moreover, TGS 121 induced changes in the tight junction complexes expression in the intestinal wall. This is the first study proving that gold (III) complexes may have therapeutic potential in the treatment of IBD.

## 1. Introduction

The biomedical potential of gold has been discussed in numerous reports, concentrating both on its nanoparticle [1,2,3,4,5] and complex forms. The main application of gold nanoparticles (AuNPs) is cancer diagnostics and therapy and, as a new concept, “theranostics”, where one system serves both functions [1]. In cancer therapy, AuNPs may be utilized in various ways, including photothermal and radiofrequency therapies [4]. Another important use of AuNPs is as drug carriers, where they have many beneficial features: they are non-toxic and non-immunogenic, highly permeable and accumulate at the tumor sites [3]. AuNPs have potential in inflammatory diseases as well [6,7,8]. 

Gold has also been studied in the form of complexes, and various gold (I) and gold (III) derivatives have been tested as potential anticancer drugs [9,10]. There has been particular interest in the therapeutic efficacy of gold (I) complexes, which, similarly to gold (III) complexes , have been found to inhibit selenium and sulfur-containing enzymes such as thioredoxin reductase (TrxR), glutathione peroxidase, cysteine protease, and glutathione-S-transferase, and therefore cause apoptosis [9]. For example, in a study by Gomes et al., a new gold (I) complex was tested in nine tumorigenic and one non-tumorigenic cell line, and showed high selectivity towards ovarium adenocarcinoma (OVCAR-3) [11]. Marmol et al. tested an alkynyl gold (I) complex on the colorectal adenocarcinoma Caco-2 cell line [12]. This compound showed an anticancer activity, as it triggered necroptosis, which could provide a treatment option in cases of apoptosis resistance [12]. In another study, gold (I)-coumarin-caffeine-based complexes were tested as potential anticancer and anti-inflammatory agents [13]. They showed antiproliferative properties on breast, prostate, and colon cancer cell lines. Two complexes showed anti-inflammatory effects, evaluated through the inhibition of IL-1β production by peripheral blood mononuclear cells, while one displayed a pro-inflammatory behavior. The anti-inflammatory effect was altered by the introduction of the coumarin moiety, highlighting the importance of the ligands and their position on the properties of the complex [13]. 

The analogy between platinum (II) and gold (III), both of which have a d8 electronic configuration, led to the belief that gold (III) compounds may have anticancer properties like platinum-based metallodrugs, such as cisplatin or oxaliplatin [14,15], which was further verified in preclinical studies. A gold (III) porphyrin complex (Au (TPP)Cl) displayed cytotoxicity in various cancer cell lines and showed potential in vivo as an anticancer agent in nasopharyngeal carcinoma, hepatocellular carcinoma, colon cancer, neuroblastoma, and melanoma. A gold (III) complex with tridentate C-deprotonated C^N^C ligand showed anticancer properties in vitro and in animal models of hepatocellular carcinoma and sarcoma. Other groups studied for their anticancer activity constitute organogold (III) complexes with C-deprotonated C^N and C^N^N ligands, gold (III) complexes with other multidentate N-donor ligands and gold (III)–dithiolcarbamate complexes [15]. However, it needs to be mentioned that two different modes of anticancer action for gold (III) complexes have been proposed: they could act similarly to Pt (II) (intercalation) or by targeting enzymes, e.g., thioredoxin reductase [15].

Although the beneficial properties of gold have been suspected for years and its compounds have been tested in various diseases, they are currently in clinical use only against rheumatoid arthritis [9]. However, auranofin, a gold(I)-containing compound registered as a drug against rheumatoid arthritis, has also shown anticancer activity in animal models and is approved for clinical trials for lung and ovarian carcinoma [16]. What is more, auranofin has also been shown to possess antibacterial and antivirulence activities against Clostridium difficile [17]. Auranofin inhibited *Clostridium difficile* toxin production and spore formation, showing direct protective activity against toxin-mediated inflammation [17]. Another study carried out by the same group showed the potential of auranofin for use as a decolonizing agent for vancomycin-resistant enterococci [18]. Auranofin showed a potent antimicrobial activity against multiple enterococcal clinical isolates. Importantly, no resistant mutants were developed over the course of 14 passages [18]. 

In this study we present a novel gold (III) complex, TGS 121, of the following structure: [Au(CN)_4_]_2_ (ClO_2_)Na. We examined its anti-inflammatory properties in vitro in lipopolysaccharide(LPS)-stimulated RAW264.7 macrophages, and in vivo in murine dextran sulphate sodium (DSS)-induced model of colitis. We investigated the molecular mechanism of its action through modulation of antioxidant mechanisms and the influence on tight junction proteins.

## 2. Results

### 2.1. Characterization of TGS 121

Physicochemical studies showed that TGS 121 was highly water-soluble; it was stable in water, blood, and lymph, and impervious to sunlight.

### 2.2. TGS 121 Showed an Anti-Inflammatory Profile In Vitro in RAW264.7 Macrophages

The in vitro studies consisted of a neutral red uptake (NRU) test to verify the cytotoxicity of the compound and a Griess test to assess the NO production.

**Figure 1:**
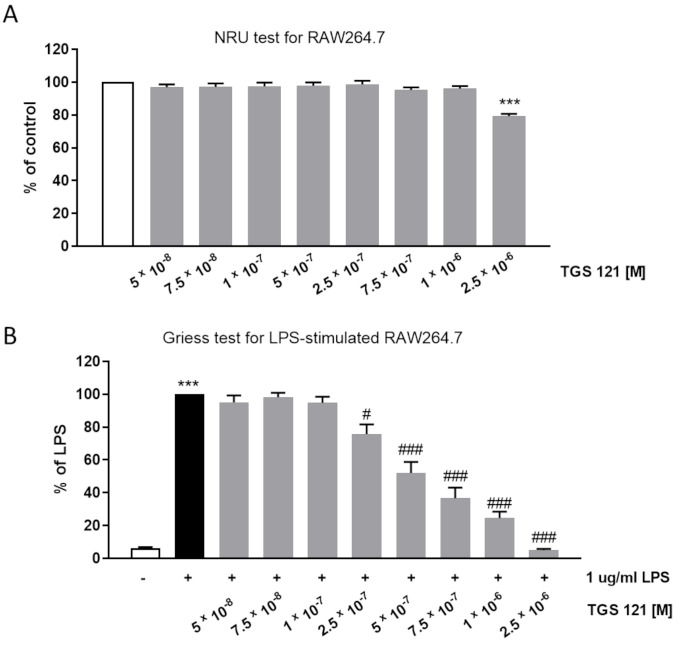
Neutral red uptake test (**A**) and Griess test (**B**) for RAW264.7 macrophages treated with TGS 121. *** *p* < 0.001 vs. control; # *p* < 0.05, ### *p* < 0.001 vs. 1 ug/mL lipopolysaccharide (LPS).

The NRU test showed no decrease in viability up to 1 × 10^−6^ M (Figure 1A). As the next step, we assessed how the NO production in RAW264.7 was influenced by the treatment with TGS 121. In the Griess test, NO production is evaluated based on the concentration of nitrite in cell culture supernatants. The efficacy of the studied compound is assessed at concentrations which do not cause decrease in viability. We observed that NO production was significantly decreased by TGS 121 at the concentration 2.5 × 10^−7^ (down to 75.80 ± 5.89%, where 100% is the value for untreated, LPS-stimulated cells), and the inhibition was concentration-dependent (52.07 ± 6.67% at 5 × 10^−7^, 36.70 ± 6.40 at 7.5 × 10^−7^, and 24.53 ± 3.91% at 1 × 10^−6^ M) (Figure 1B).

### 2.3. TGS 121 Attenuated Colitis in DSS-Induced Mouse Model

In the DSS-induced mouse model of colitis, TGS 121 was tested in two doses: 1.68 μg/kg (A), intragastrically , and 16.8 μg/kg (B), intragastrically , once daily from day 3 to day 6 after colitis induction. 

**Figure 2:**
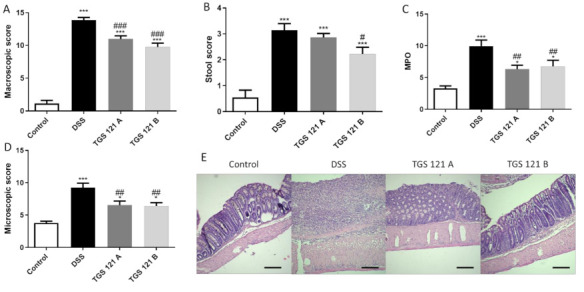
The effect of TGS 121 on colonic inflammation in a dextran sulphate sodium (DSS)-induced model of colitis: macroscopic score (**A**), stool score (**B**), myeloperoxidase activity (**C**), and microscopic score (**D**) for control, DSS-only treated mice, and mice with DSS-induced colitis treated with TGS 121 in two doses: 1.68 μg/kg (TGS 121 A) and 16.8 μg/kg (TGS 121 B). Representative photos of hematoxylin and eosin staining of colon samples (**E**). Scale bar = 100 μm. *p < 0.05 and *** *p* < 0.001 as compared with the control mice; # *p* < 0.05, ## *p* < 0.01, ### *p* < 0.001 as compared with DSS-treated animals.

DSS treatment induced colitis, as evidenced by the increased macroscopic score (1.15 ± 0.47 for control mice vs. 13.85 ± 0.42 for DSS-only treated animals) (Figure 2A), which is consistent with our previous observations [19]. TGS 121 significantly decreased the macroscopic score: it equaled 10.98 ± 0.49 for 1.68 μg/kg and 9.75 ± 0.61 for 16.8 μg/kg compared to DSS-only treated mice (*p* < 0.001 in both cases) (Figure 2A). Similarly, stool score was increased for DSS-only treated mice (0.55 ± 0.28 for control vs. 3.15 ± 0.25 for DSS-treated animals), and this effect was reversed by treatment with 1.68 μg/kg TGS 121 (2.86 ± 0.15) and 16.8 μg/kg TGS 121 (2.23 ± 0.26, *p* < 0.05) (Figure 2B).

The myeloperoxidase activity represents neutrophil infiltration of the intestine, therefore it is highest in inflamed tissue. The activity was lowest for control mice (3.32 ± 0.38 U). The activity was highest in the DSS-only treated mice (9.11 ± 1.30 U), while it was lower for TGS 121-treated animals (6.33 ± 0.61 U, *p* < 0.01 for 1.68 μg/kg, and 7.64 ± 1.06 U, *p* < 0.01 for 16.8 μg/kg) (Figure 2C). 

The parameters taken into consideration during microscopic evaluation included muscle thickness, cell infiltration, mucosal architecture, crypts morphology, and the presence of Goblet cells. DSS treatment significantly increased microscopic score (9.25 ± 0.68 for DSS-only treated animals vs. 3.80 ± 0.26 for control, *p* < 0.001). TGS 121 in both concentrations caused statistically significant decrease in microscopic score (Figure 2D), (6.55 ± 0.62 for 1.68 μg/kg, *p* < 0.01 and 6.40 ± 0.52 for 16.8 μg/kg, *p* < 0.01).

### 2.4. TGS 121 Influenced the Antioxidant Profile in the Mouse Colon

**Figure 3:**
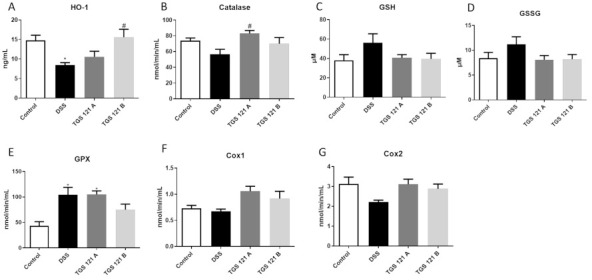
The influence of DSS colitis induction and TGS 121 treatment on the antioxidant profile in the mouse colon: heme oxygenase-1 (**A**), catalase (**B**), glutathione (**C**), glutathione disulfide (**D**), glutathione peroxidase (**E**), cyclooxygenase-1 (**F**), and cyclooxygenase-2 (**G**) for control, DSS-only treated mice, and mice with DSS-induced colitis treated with TGS 121 in two doses: 1.68 μg/kg (TGS 121 A) and 16.8 μg/kg (TGS 121 B). * *p* < 0.05 as compared with the control mice; # *p* < 0.05 as compared with DSS-treated animals.

In DSS-only treated mice heme oxygenase-1 (HO-1) level was decreased compared to control (*p* < 0.05). Treatment with 1.68 μg/kg TGS 121 slightly increased HO-1 level in DSS-treated animals, while for 16.8 μg/kg the difference was significant (*p* < 0.05) (Figure 3A). Similarly, DSS treatment decreased the activity of catalase; TGS 121 increased catalase activity and for 1.68 μg/kg the difference was significant (*p* < 0.05) (Figure 3B). 

DSS administration slightly increased the level of glutathione (GSH) and glutathione disulfide (GSSG) in colonic tissue, and this effect was reversed by TGS 121 (Figure 3C and Figure 3D, respectively). Glutathione peroxidase (GPX) activity was significantly increased (*p* < 0.05) by DSS treatment, but the increase was diminished when 16.8 μg/kg TGS 121 was administered (Figure 3E).

Cyclooxygenase-1 (COX-1) activity was not influenced by the DSS treatment, however, after TGS 121 administration COX-1 activity increased (Figure 3F). Cyclooxygenase-2 (COX-2) activity was slightly decreased in the DSS group, but TGS 121 administration reversed this effect (Figure 3G).

### 2.5. TGS 121 Induced Changes in the Tight Junction Complexes Expression

**Figure 4:**
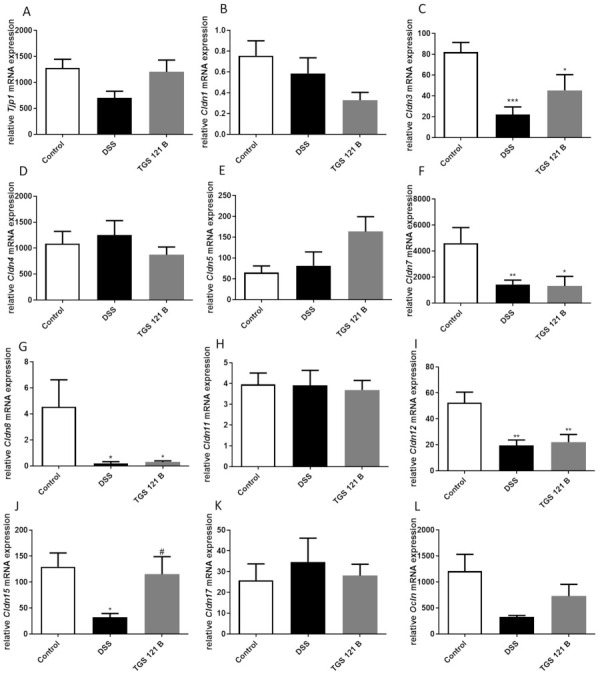
The influence of DSS colitis induction and TGS 121 treatment on the expression of intestinal wall function proteins for control, DSS-only treated mice, and mice with DSS-induced colitis treated with TGS 121 at 16.8 μg/kg (TGS 121 B): Tjp1 (**A**), Cldn1 (**B**), Cldn3 (**C**), Cldn4 (**D**), Cldn5 (**E**), Cldn7 (**F**), Cldn8 (**G**), Cldn11 (**H**), Cldn12 (**I**), Cldn15 (**J**), Cldn17 (**K**), Ocln (**L**).* *p* < 0.05, ** *p* < 0.01, *** *p* < 0.001 as compared with the control mice; # *p* < 0.05 as compared with DSS-treated animals.

The expression of genes for proteins associated with the intestinal barrier function was assessed in the mouse colon samples at the mRNA level. The higher dose of TGS 121 was selected for these studies. We observed a decrease in Tjp1, Cldn3, Cldn15, and Ocln mRNA levels in DSS-treated mice compared with control animals; of note, the effect was statistically significant for Cldn3 and Cldn15 (*** *p* < 0.001 and * *p* < 0.05, respectively) (Figure 4A,C,J,L). For all four genes, administration of 16.8 μg/kg TGS 121 increased respective mRNA levels; in the case of Cldn15 a significant change (# *p* < 0.05 vs. DSS-treated mice) was obtained. The expression of Cldn7, Cldn8, and Cldn12 significantly decreased in DSS-treated mice compared with controls (** *p* < 0.01, * *p* < 0.05 and ** *p* < 0.01, respectively), and no effect of TGS 121 was shown (Figure 4F,G,I). The mRNA levels for Cldn1, Cldn4, Cldn5, Cldn11, and Cldn17 did not change in the mouse colon after DSS treatment compared with controls (Figure 4B,D,E,H,K). For Cldn1, the treatment with TGS 121 non-significantly decreased the gene expression compared with samples from animals with colitis; in contrast, the expression of Cldn5 increased; in the case of all other genes there were no differences in expression at the mRNA level between groups.

## 3. Discussion

Despite the therapeutic potential of gold (I) and gold (III) complexes, reports concerning their anti-inflammatory properties are very scarce. Various gold (I) complexes have shown anti-inflammatory effects in vitro on LPS-activated THP-1 macrophages, and in vivo, in the carrageenan-induced hind paw edema model on rats [20,21]. In other studies the same models were used to test a series of gold (III) complexes involving differently substituted derivatives of a plant hormone, N6-benzyladenine [22], and cycloaurated phosphine sulfide complexes [23]. However, until now the potential of gold complexes in colitis has not been studied. 

In our study we showed the anti-inflammatory potential of a new gold (III) complex, TGS 121. The anti-inflammatory activity was documented in vitro as well as in the mouse model of DSS-induced colitis through macroscopic, stool, and microscopic scores, and MPO activity. We also analyzed the mechanism of action of TGS 121 and found that it acts through modulation of the antioxidant enzymatic and non-enzymatic system and tight junction protein expression. 

In physiological conditions the concentration of reactive oxygen species (ROS) and reactive nitrogen species (RNS) is maintained at a non-harmful level thanks to anti-oxidative defense mechanisms, which comprise enzymes such as CAT, SOD, and GPx, and non-enzymatic scavengers like glutathione (GSH) [24]. However, inflammatory reactions lead to an excessive release of free radicals from leukocytes and activated macrophages, which results in oxidative and nitrosative stress [24,25]. Free radicals can damage both nuclear and mitochondrial DNA, RNA, lipids, and proteins [26]. 

Heme oxygenase (HO) is an enzyme which shows anti-inflammatory activity, and its induction can potentially be used in the treatment of inflammatory disorders [27]. There are two isoenzymes of HO, of which HO-1 is the inducible isoform. HO-1 is not a direct catalyst involved in antioxidant reaction, its induction is regarded as an adaptive cytoprotective response against oxidative stress [27]. In a study by Berberat et al. HO-1 was upregulated by the administration of cobalt-protoporphyrin, and it attenuated mucosal injury, delayed diarrhea and gastrointestinal hemorrhage, and decreased body weight loss [28]. Similarly, induction of HO-1 in *IL10-/-* mice reduced development of colitis [29]. Onyiah et al. concentrated on the influence of modulating HO-1 expression on the inflammatory response of human intestinal epithelial cells (IECs) [30]. They observed that the deficiency of HO-1 in IECs resulted in increased proinflammatory chemokine expression, while the treatment of cells with an inducer of HO-1 inhibited this process, which suggests that HO-1 is a central regulator of IEC chemokine expression [30]. In our study we observed a significant decrease in HO-1 level in the tissues of DSS-treated mice compared with control animals. Amelioration of colitis after treatment with TGS 121 was accompanied by an increase in HO-1 concentration (the difference in HO-1 level was significant for the higher dose tested), confirming that induction of HO-1 results in alleviation of inflammation, which is in line with other studies.

Catalase is another enzyme which belongs to the endogenous enzymatic antioxidant system. It is crucial for regulating H_2_O_2_ levels, as it catalyzes the decomposition of H_2_O_2_ to H_2_O and O_2_ [31]. Pervin et al. reported significantly lower CAT activity in a DSS-induced murine colitis group compared to the control group, while treatment with blueberry extract, which attenuated colitis, resulted in elevation in CAT activity levels [32]. In acetic acid-induced colitis in rats CAT activity was also reduced as compared to the healthy control [33]. Similarly, in DSS-induced colitis in rats CAT activity was reduced, and with the administration of treatment (tyrosol) it came to the same level as the control group [34]. The above-mentioned results are well in-line with our study. We also observed a decrease in CAT activity in the DSS-treated group, while the administration of TGS 121 caused its significant increase.

GSH is often seen as the most important intracellular antioxidant, and the GSH-GSSG-GPx system may be regarded as the most crucial in maintaining cellular homeostasis. In the presence of reactive oxygen species GSH undergoes oxidation to glutathione disulfide (GSSG), catalyzed by GSH peroxidase (GPx) [35]. In acetic acid-induced ulcerative colitis in rats and mice, colonic levels of glutathione decreased in the colitis group compared to control [36,37]. Similarly, in a DSS-model of colitis in male Wistar rats, DSS significantly reduced GSH levels in the colon [34,38], while treatment with allopurinol, sulfasalazine, and febuxostat elevated GSH content [38]. In contrast to the described findings, in our study, we observed an insignificant increase in GSH level in the DSS-treated group, while TGS 121 treatment reversed this effect. Further investigations are needed to verify these observations.

GSSG is increased in the inflamed tissue of inflammatory bowel diseases (IBD) patients [35]. Moreover, Anzoise et al. reported increased GSSG level in acetic acid induced colitis in rats [39]. In line with those reports, we observed a slight increase in GSSG level in the DSS-treated mice and normalization to the level of healthy control after the TGS 121 treatment.

In a murine model of T cell transfer colitis, inflammation decreased colon GPx activity [40]. Similarly, in a rat DSS model of colitis, GPx activity was significantly reduced in the group given DSS, compared to control [34]. In contrast to those findings, Kruidenier et al. reported an increased activity of GPx in IBD patients mucosae [41]. In our study, DSS treatment caused significant increase in GPx activity, and the higher dose of TGS 121 reversed this effect.

There are two basic isoforms of cyclooxygenase (COX), COX-1 and COX-2. COX-1 is constitutively expressed in most tissues, and has been proposed to maintain the cell integrity of the gastrointestinal tract, while COX-2 is not produced in substantial amounts in epithelial cells, but is rapidly induced in inflammatory sites and expressed in colonic adenomas, carcinomas, and IBD [42,43]. Peng et al. reported reduced COX-1 mRNA and protein expression in colonic specimens obtained from patients with UC [42]. Similarly, in DSS-treated mice COX-1 mRNA levels and protein levels were decreased [42]. Similarly, we observed a decrease, although minor, in COX-1 activity between the control and DSS groups, and its non-significant increase after TGS 121 treatment. Increase in COX-2 expression has been observed in colon mucosal samples from UC patients [42] and in animal models of colitis [33,34,44,45]. Surprisingly, we observed a slight decrease in COX-2 activity in the DSS group, and this effect was reversed by the treatment with TGS 121, which requires further studies.

We also tested the effect of TGS 121 on the expression of a number of tight junction proteins: Tjp1, claudin 1, 3, 4, 5, 7, 8, 11, 12, 15, and 17, and occludin. While DSS treatment decreased the expression level of Tjp1, as reported by Luo et al. [46], TGS 121 reversed this effect. Claudin 1 expression has been reported to rise or not to change in colitis [47,48]. In our case, the expression stayed at the same level in the DSS-treated group, compared with control, but was slightly decreased after treatment with TGS 121. According to Ahmad et al. claudin 3 expression was lower both in IBD and a DSS-induced mouse model of colitis [48]. We also observed a significant decrease in the DSS-treated group, while the administration of TGS 121 reversed this effect. The expression of claudin 5 did not change after DSS treatment, consistent with the literature [47,48], however, the treatment with TGS 121 slightly increased claudin 5 mRNA level. Claudin 7 expression decreased in UC, as well as a DSS model of colitis [48], which is consistent with our results, but TGS 121 did not influence its expression. Claudin 8 and claudin 12 had decreased expression in colitis, consistent with the literature [47,48], and TGS 121 treatment had no influence. Occludin was reported to be downregulated in colitis and, consistently, in our study its expression was reduced in the DSS group. Treatment with TGS 121 slightly increased occludin mRNA level.

Of note, the expression of claudin 15 has not yet been reported in patients with IBD. In our study, DSS treatment caused a statistically significant decrease in claudin 15 mRNA level, and administration of TGS 121 significantly increased its expression. In this study we showed that claudin 15 may have importance in IBD pathogenesis, moreover, it is modified by TGS 121.

In conclusion, here we report on TGS 121, the first representative of a new class of gold (III) compounds with anti-inflammatory activity in colitis upon oral administration. Our study gives a very solid basis for further pre-clinical investigations of this gold (III) complex as an attractive therapeutic option for inflammation, and a good starting point for the synthesis of novel derivatives, as well as promising clinical translation. This is the first representative of a new class of compounds, therefore more studies are needed to locate its efficacy and therapeutic potential among conventionally used drugs. It is unclear whether the observed changes in the antioxidant profile and the tight junction complex expression are a cause or a consequence of TGS 121 treatment, therefore the proposed mechanisms of action require further verification. Other issues, such as different putative mechanisms of action or the determination of the therapeutic window, should also be taken into consideration in further studies.

## 4. Materials and Methods

### 4.1. Synthesis of TGS 121

The chlorite-cyanide complex of monoionic gold (III) was prepared as follows: 1000 mg of 99.99% pure metallic gold was dissolved in a mixture of concentrated hydrochloric and nitric acid in a molar ratio of 3:1. After dissolution, gold (III) appeared in the form of very large clusters with metallic bonds (Au-Au)>11. The water-soluble gold (III) clusters were acidified with 350 cm^3^ of concentrated (36%) aq hydrochloric acid, then the mixture was boiled until the volume decreased to 20–30 cm^3^. After adding 350 cm^3^ of concentrated HCl, the solution was heated again to the boiling point until the nitrosyl chloride (NOCl) vapors were released. The above operation was repeated many times until the nitric acid and its oxides were completely evaporated and the gold (III) chlorides remained in the flask.

A thermostated polyglycol bath was used to evaporate the liquid (acids) from the gold (III) salt. The dry salts obtained as described were redissolved in aqua regia. The above chemical treatment enables the preparation of clusters of gold (III) chloride smaller than 11-atom.

Then, 450 cm^3^ of 6 M HCl was added to the dry salt; the mixture was heated again to the boiling point of the liquid and evaporated until dry salt formed. This operation was repeated four times in order to obtain the smallest possible gold (III) clusters. An orange-red salt of gold (III) chloride was obtained, the analysis of which showed the presence of practically pure Au_2_Cl_6_.

Next, 9 g of sodium chloride (NaCl) were added to the obtained Au_2_Cl_6_ (the molar ratio of NaCl to gold over 300) and topped up with distilled water to approx. 500 cm^3^. Then the mixture was boiled for several hours to obtain a compound with the formula Na_2_Au_2_Cl_8_.

The aqueous solution of sodium chloride and salt was evaporated until the salt was dry. Then the salts were treated alternately with 400 cm^3^ of distilled water and 600 cm^3^ of 6M HCl, until no further color change was visible.

After the last treatment with 6M HCl and its final evaporation, dry salts were obtained, which were then diluted with 800 cm^3^ of distilled water, obtaining a solution of HAuCl_2_*H_2_O monatomic gold salt with a pH of approximately 1.0.

Next, 1M NaOH was added into the flask to neutralize the solution to pH 4–5, then 3 g of 25% solution of NaClO_2_ were added. After several hours, a water-soluble, stable complex of gold (III) with chlorine dioxide and sodium chloride was obtained, with the formula: NaAuCl_4_*ClO_2_*(NaCl)_z_, where z is a number over 30.

The mono-ionic NaAuCl_4_*ClO_2_*(NaCl)_2_ complex was neutralized with 5% NaOH to a pH of about 7.8, and then 60 g of 0.5M aqueous-alcoholic sodium cyanide solution was added. The mixture was stirred at 35 °C for 2 h, and then acidified with 0.2 M HCl under a fume hood. The above mixture was agitated in vacuo for 4 h to drive off free hydrogen cyanide. Well-soluble in water complexes of mono-ionic gold (III) were neutralized to pH 7.4 with 0.1M NaOH. Next, redistilled water was added to the volume of 1 dm^3^.

The subject of synthesis was diluted tenfold with saline (9 g/dm^3^ NaCl) and was termed TGS 121. The structure was confirmed by the FT-IR method. The purity of the sample was confirmed by the ICP-MS method, according to Ph. Eur. 2.2.58 monograph: calculated 0.231 mg/dm^3^ for the dose 1.68 µg/kg, and 2.31 mg/dm^3^ for the dose 16.8 µg/kg, respectively; found 0.229 and 2.30 mg/dm^3^, respectively.

### 4.2. Other Drugs and Reagents

Cell culture media and media supplements were purchased from Gibco (Waltham, MA, USA). All other drugs and reagents, unless stated otherwise, were purchased from Sigma-Aldrich (Poznań, Poland).

### 4.3. Cell Line Culture

The RAW264.7 murine macrophage cell line was obtained from the American Type Culture Collection (ATCC TIB-71). RAW264.7 cells were maintained in a humidified atmosphere of 5% CO_2_ at 37 °C. Cells were grown in Dulbecco’s modified eagle medium (Gibco, Waltham, MA, USA) supplemented with 10% BCS, 2 mM Ala-Gln, 0.5% P/S, 1 mM sodium pyruvate, and 25 mM HEPES. Culture medium was replaced every two or three days, and the cells were trypsinized after reaching approx. 80% confluence.

### 4.4. Cytotoxicity Assessment

The cytotoxicity of TGS 121 on RAW264.7 cell line was assessed with the use of a neutral red uptake (NRU) assay after 48 h of exposure to the studied compound. The complex was tested in the range of concentrations: 5 × 10^−8^ – 2.5 × 10^−6^ M.

The assay is based on the staining of living cells by neutral red (NR), which represents the lysosomal activity of the cells. The experiments were carried out according to the protocol described previously [19]. The cells were seeded on 96-well plates at the density of 20,000/well and incubated for 48 h with or without the studied complex. After this incubation, the medium was removed and 100 µL/well of 0.05 mg/mL NR solution in culture medium was added. After 1 h incubation, the cells were washed once with PBS (pH 7.4) and 100 µL/well of dye solvent (40% ethanol and 10% acetic acid in water) was added. The plates were shaken for 10 min on a rotary shaker and the absorbance was measured at 540 nm in a microplate reader (iMARK Microplate Reader, Biorad, Hertfordshire, UK) using blank as a reference. Cytotoxicity was expressed as a percentage of negative control (medium without the studied complex).

### 4.5. Griess Assay

In order to measure the nitrite concentration in cell culture supernatants a Griess assay was used, as described previously [19]. The cells were seeded on 96-well plates at the density of 20,000/well and incubated for 24 h with medium only (control) or 1 µg/mL LPS with or without the studied complex (in the same concentrations as for cytotoxicity assessment). After 24 h, the medium was removed and solutions containing TGS 121, without LPS, were added. After another 24 h, 40 mg/mL Griess reagent water solution was mixed with an equal volume of cell culture supernatant. The mixture was incubated for 15 min in the dark, and the absorbance was read at 540 nm.

### 4.6. Animals

Male balbC mice were obtained from the Animal Facility of University of Lodz, Poland. The animals used in the experiments weighed 22–27 g (approx. 8 weeks of age). The animals were housed at a constant temperature (22–23 °C) and maintained under a 12-h light/dark cycle with free access to laboratory chow and tap water. Animal protocols were approved by the Local Ethical Committee for Animal Research at the Medical University of Lodz (#4/ŁB85/2018 from 15 January 2018 and #13/ŁB130/2019 from 15 April 2019). All efforts were made to minimize animal suffering and to reduce the number of animals used.

### 4.7. Induction of Colitis

Colitis was induced by administration of dextran sulfate sodium (DSS; molecular weight 40,000, Biochemica, PanReac AppliChem, Darmstadt, Germany), as described earlier [19]. Mice (n = 8–10 per experimental group) received DSS (4% *wt*/*vol*) in drinking water from day 0 to day 5. On days 6 and 7 the animals received water. Control mice were given drinking water throughout the whole experiment. Animal body weight was monitored daily. Mice were sacrificed by cervical dislocation on day 7 and colonic damage was evaluated.

### 4.8. Pharmacological Treatment

TGS 121 was administered intragastrically once daily from day 3 to day 6 after colitis induction, 100 µL/mouse, at the doses of 1.68 μg/kg (A) and 16.8 μg/kg (B). Control and DSS groups received 0.9% NaCl alone (100 μL, intragastrically).

### 4.9. Evaluation of Colonic Damage

For total macroscopic score evaluation, the colon was removed immediately after cervical dislocation and weighed (including fecal content). Next, the colon was opened longitudinally and the feces were removed. The well-established semiquantitative scoring system used took into consideration the following parameters: colon length and weight scores (0–4), stool consistence (0–3), and colon epithelial damage, that is, number of ulcers (0–3), where score = 0 means no inflammation. The presence (score = 1) or absence (score = 0) of fecal blood was also recorded. The macroscopic scoring was performed in a blind manner.

### 4.10. Microscopic Score Evaluation

Distal colon sections (approx. 0.5 cm in length) were stapled flat, mucosal side up, onto cardboard and fixed in 10% neutral-buffered formalin for at least 24 h at 4 °C. Next, samples were dehydrated in sucrose and embedded in paraffin, sectioned at 5 μm and mounted onto slides. The hematoxylin-eosin stained sections were examined using a Zeiss Axio Imager setup (Jena, Germany). Microscopic total damage score was determined based on the following parameters: presence (score = 1) or absence (score = 0) of goblet cell depletion, the presence (score = 1) or absence (score = 0) of crypt abscesses, the destruction of mucosal architecture (normal = 1, moderate = 2, extensive = 3), the extent of muscle thickening (normal = 1, moderate = 2, extensive = 3), and the presence and degree of immune cell infiltration (normal = 1, moderate = 2, transmural = 3).

### 4.11. Determination of Tissue Myeloperoxidase Activity

In order to monitor the degree of inflammation, myeloperoxidase (MPO) activity was determined using a standardized method, as described earlier [19]. Briefly, colon segments (approx. 30 mg) were homogenized in hexadecyltrimethylammonium bromide (HTAB) buffer (0.5% HTAB in 50 mM potassium phosphate buffer, pH 6.0; 50 mg tissue/mL). Homogenates were centrifuged (15 min, 13,200× *g*, 4 °C). On a 96-well plate, 200 μL of 50 mM potassium phosphate buffer (pH 6.0), supplemented with 0.167 mg/mL of O-dianisidine hydrochloride and 0.05 μL of 1 % hydrogen peroxide, was added to 7 μL of the supernatant. Absorbance was measured at 450 nm after 30 and 60 s (iMARK Microplate Reader, Biorad, Hertfordshire, UK). All measurements were performed in triplicate. MPO activity was expressed in milliunits per gram of wet tissue, 1 unit being the quantity of enzyme able to convert 1 μmol hydrogen peroxide to water in 1 min at room temperature.

### 4.12. Oxidative Measurements

Biomarkers of oxidative stress (Catalase, GSH, GSSG, GPX, Cox1 and Cox2) from the murine colonic samples were assessed by the commercial antioxidant enzyme assay kits (Cayman Chemical Company, Ann Arbor, MI, USA; Item numbers: 707002, 703002, 703102, 700200, 709001). Level of mouse Hemooxygenase-1 was determined by the HO-1 ELISA Kit (EIAab kit #E0584m, Wuhan, China). Tissue specimens were collected and homogenized according to the manufacturer’s instructions. Each assay was performed following the provided user’s manual and each sample was assayed in duplicate.

### 4.13. Assessment of Tight Junction Complexes Expression

#### 4.13.1. RNA Isolation

RNA extraction was performed using murine colon samples and commercially available EXTRAzol reagent (Blirt, Gdańsk, Poland) in accordance with the manufacturer’s protocol. The purity and quantity of RNA was estimated spectrophotometrically with BioPhotometer Plus (Eppendorf, Hamburg, Germany). The RNA was characterized with A_260_ nm/A_280_ nm ratio, which was in the range of 1.7–2.0.

#### 4.13.2. Reverse Transcription and Real-Time PCR

cDNA synthesis was performed with a High-Capacity cDNA Reverse Transcription Kit (Applied Biosystems, Waltham, MA, USA), in accordance with the manufacturer’s protocol. Then, 1 µg of RNA was used in a reverse transcription reaction with the following incubation steps: 25 °C; for 10 min, 37 °C; for 120 min, and 85 °C; for 5 min. Quantification of mRNA expression was performed using the real-time PCR method with predesigned KiCqStart^®^ primers (Sigma Aldrich, Munich, Germany), which are shown in Table 1. The reaction mixture consisted of cDNA, a PowerUp^TM^ SYBR^TM^ Green Master Mix (Applied Biosystems, Waltham, MA, USA), and forward and reverse KiCqStart^®^ primers, as well as RNase-free water in total volume of 10 µL. Cycle parameters for the real-time PCR were as follows: UDG activation at 50 °C; for 2 min and Dual-Lock^TM^ DNA polymerase at 95 °C; for 2 min, followed by 40 cycles of sequential incubations at 95 °C; for 15 s, at 57 °C; for 15 s, and at 72 °C; for 1 min. All experiments were performed in triplicate and the obtained results were normalized to the expression of housekeeping gene, that is, hypoxanthine phosphoribosyltransferase 1 (Hprt1). The reaction was performed using a Mastercycler^®^ep Realplex^4^s (Eppendorf, Hamburg, Germany). The fluorescent dye emission was a function of the cycle number. The initial amount of the temple was evaluated as a Ct parameter. Ct value corresponded to the threshold cycle number at which PCR amplification reached a significant threshold. The relative expression level was calculated as 2^−∆Ct^ × 1000 and the results are expressed as the number of examined mRNA copies per 1000 copies of mRNA for Hprt1.

### 4.14. Statistical Analysis

The statistical analysis was performed in Prism 5 (GraphPad Software Inc., La Jolla, CA, USA) with the use of one-way ANOVA followed by Newman–Keuls post hoc test. The data are presented as mean ± standard error of the mean (SEM). *p* values < 0.05 were considered statistically significant.

## Figures and Tables

**Table 1:** Primer sequences of tight junction genes and the housekeeping gene.

Gene	Primer	Sequence
*Cldn1*	Forward	TTTTAATTTCAGGTCTGGCG
	Reverse	CAAATTCATACCTGGCATTG
*Cldn3*	Forward	ATTTCTATAACCCGTTGGTG
	Reverse	AGAATAGAGGATCTTGGTGG
*Cldn4*	Forward	GACTGTGCAAAGTTACTAGC
	Reverse	ACCAGCAATTTGGATGTAAG
*Cldn5*	Forward	AACAGTTCCTACTGAGATCC
	Reverse	CTTTTTAACACGTCCCTCTG
*Cldn7*	Forward	CATTGTTTTCATTGTGGCAG
	Reverse	CTCGTACTTAACGTTCATGG
*Cldn8*	Forward	GAAGTCATATCATGCAAGGG
	Reverse	TGAGTGTCAGGAGTTAGAAG
*Cldn11*	Forward	CTGGTGGACATCCTCATC
	Reverse	AGAGAGCCAGCAGAATAAG
*Cldn12*	Forward	ATTATCTCTGCTTTGTGTGC
	Reverse	TTCAAGGTAATCAGCGTTTC
*Cldn15*	Forward	ACATGGATCTCTCCAAGAAG
	Reverse	CATACTTGGTTCCAGCATAC
*Cldn17*	Forward	TCGGCAGCAACATTATTATC
	Reverse	ATGCCAATAATGAGAGCAAC
*Ocln*	Forward	GGAGAGTGAAGAGTACATGG
	Reverse	TGTCATAATCTCCCACCATC
*Tjp1*	Forward	ACTAGCATCAGACCATTCAG
	Reverse	CGTCTACATGTTTTACAGGAG
*Hprt1*	Forward	AGGGATTTGAATCACGTTTG
	Reverse	TTTACTGGCAACATCAACAG

## Data Availability

The data presented in this study are available on request from the corresponding author.
